# Identification of Quantitative Trait Loci and Candidate Genes Involved in Rice Seedling Growth Under Hypoxic Stress

**DOI:** 10.3390/ijms262110420

**Published:** 2025-10-27

**Authors:** Nari Kim, Rahmatullah Jan, Saleem Asif, Sajjad Asaf, Zakirullah Khan, Kyung-Min Kim

**Affiliations:** 1 Department of Applied Biosciences, Kyungpook National University, Daegu 41566, Republic of Korea; jennynari@hanmail.net (N.K.); zakirullah371@gmail.com (Z.K.); 2 Coastal Agriculture Research Institute, Kyungpook National University, Daegu 41566, Republic of Koreasaleemasif10@gmail.com (S.A.); 3 Natural and Medical Science Research Center, University of Nizwa, Nizwa 616, Oman; sajadasif2000@gmail.com

**Keywords:** rice cultivars, hypoxia tolerance, quantitative trait loci (QTL), gene expression, stress response

## Abstract

Studying hypoxia in rice is particularly important because oxygen deficiency during germination severely limits seedling establishment. Understanding the molecular and physiological mechanisms underlying hypoxic tolerance is therefore crucial for improving rice yield stability under flooded or waterlogged conditions. Progress in developing rice cultivars that thrive under flooding and low oxygen (hypoxic) conditions has been limited over the past two decades due to a lack of tolerant plant varieties and a limited understanding of genetic mechanisms. This study evaluated hypoxia tolerance in the Cheongcheong Nagdong Double Haploid (CNDH) rice population, along with their parent lines, for hypoxia tolerance. Significant phenotypic differences were identified, with the Cheongcheong and CNDH lines CNDH13, CNDH35, and CNDH91 showing strong hypoxia tolerance, while Nagdong and CNDH lines CNDH14-2, CNDH43, and CNDH50-1 were susceptible to hypoxia. Root length was unaffected by hypoxia, while shoot length and fresh weight were key tolerance indicators. Comprehensive quantitative trait loci (QTL) analysis based on logarithm of the odds (LOD) scores above 3.0 identified three QTLs associated with hypoxia tolerance, indicating significant genetic control: *qSL-8* and *qSL-10* (shoot length) and *qFW-2* (fresh weight). The gene expression analysis performed under hypoxic conditions highlighted that 35 candidate genes within these QTL regions exhibited differential regulation: *Os02g0184200*, *Os08g0430200*, *Os08g0431900*, and *Os08g0432500* were upregulated, whereas *Os08g0439100*, *Os10g0343400*, *Os10g0395400*, and *Os10g0405600* were downregulated in both resistant and susceptible lines. *Os08g0431900* displayed significant expression changes correlating with hypoxia resistance. Phylogenetic and protein–protein interaction analyses revealed that *Os08g0431900* is highly conserved and interacts with proteins involved in stress responses, suggesting that these proteins are crucial in hypoxia tolerance. These findings provide valuable insights into the genetic basis of hypoxia tolerance and identify key genes for future breeding programs to develop hypoxia-resistant rice varieties.

## 1. Introduction

Global warming has highlighted flooding as a serious worldwide issue, which poses a significant threat to plants, especially crops. Flooding impacts rice plant growth and photosynthesis, leading to severe yield losses [1]. Moreover, flooding can occur as waterlogging or submergence, reducing oxygen availability to plants and causing hypoxia [2]. Plant cells experience hypoxic conditions in various situations, such as waterlogged soil, flooding, dense plant tissues, seeds with gas-blocking layers, underground plant parts, or growth at high altitudes [3,4]. Subsequently, terrestrial plants have developed various adaptations to avoid severe hypoxia in their organs or enhance metabolism when oxygen is scarce [5]. For instance, rice adapts to flooding or deepwater by employing an escape strategy, producing long stems through rapid internode elongation. Additionally, rice can increase in length by 20 to 25 cm daily under flooded conditions to ensure its leaves remain above the water and normal respiration occurs [6]. Studies have shown that rice regulates phytohormones, such as ethylene, abscisic acid, and gibberellin, under deepwater conditions to enhance internode length [7,8]. Despite this rapid elongation, rice under deepwater conditions exhibits the same morphology as ordinary paddy rice [9].

In higher plants, oxygen deprivation disrupts essential cellular functions, such as mitochondrial respiration, ATP production, and overall energy supply, potentially resulting in cell death [10]. Here, the absence of oxygen means the reduced compounds generated during glycolysis and the Krebs cycle cannot be re-oxidized via the electron transport chain. Consequently, plants switch to a fermentative pathway, where NADH produced by glycolysis is re-oxidized, allowing some ATP production. During hypoxia, plants adapt by reprogramming their metabolism through the expression of specific genes that aid in overcoming adverse environmental conditions [11,12,13,14]. Waterlogging and flooding adversely affect the growth and seed production of nearly all crops, resulting in significant agricultural productivity losses and threatening food security. For instance, between 2010 and 2014, extreme monsoon rains in Pakistan led to widespread flooding, which resulted in the loss of at least 11 billion tons of crops, including rice, sugarcane, maize, and cotton, resulting in an economic losses exceeding USD 16 billion [15]. In Europe, the rise in rainfall intensity has led to numerous flooding events with severe economic impacts [16]. The detrimental effect of flooding on plant performance is primarily due to the reduction in tissue oxygen levels. Notably, rice is the only crop species capable of surviving extended periods of submergence; however, the growth and yield of rice are also affected by hypoxic conditions.

The molecular mechanisms through which plants respond to hypoxic conditions, such as flooding, submergence, and waterlogging, have been extensively studied using Arabidopsis (*Arabidopsis thaliana*) and rice (*Oryza sativa*) as model organisms [2]. Indeed, plants quickly adjust the transcription rates of various genes during changes in cellular oxygen levels. Therefore, these genes are crucial for optimizing energy use, maintaining oxygen balance, and protecting against harmful anaerobic metabolism byproducts. Rice is a semi-aquatic plant, meaning the roots of rice plants exhibit significant tolerance to hypoxic stress. MADS-box transcription factors are key regulators of various developmental and environmental responses in plants, including tolerance to abiotic stresses, such as hypoxia or submergence. Specifically, MADS-box transcription factor 23 (MADS23) plays a role in enhancing tolerance to such conditions in plants through several mechanisms. Notably, oxygen levels are limited under hypoxia or submergence conditions, meaning plants must switch from aerobic respiration to anaerobic metabolism to survive. Meanwhile, MADS-box transcription factors (TFs) have been implicated in performing key roles in adapting plants to abiotic stress. However, the underlying mechanisms through which the MADS-box proteins regulate plant stress responses remain largely unclear. MADS23 may also modulate hormone signaling pathways that are important for stress responses, such as ABA and ethylene. Moreover, MADS23 can regulate ABA-responsive genes, promoting stomatal closure, reducing water loss, and enhancing survival under stress conditions. A recent study reported that overexpression of the *OsMADS23* TF in rice plants significantly conferred osmotic stress [17]. Xingxing Li et al. (2021) further noted that the *OsMADS23* TF enhanced the indigenous ABA and proline biosynthesis pathways by regulating *OsNCED2*, *OsNCED3*, *OsNCED4*, and *OsP5CR*, which are the main components in these processes [17]. Several studies have reported that ABA is a key hormone in plant growth regulation, development, seed germination, seed dormancy, seed longevity, and seedling establishment [18,19,20,21]. The MADS23 TF induces ABA, which results in stomata formation on submerged leaves, thus controlling stomatal movement by regulating the size of guard cells and finally mediating water potential in plants [21,22,23]. Meanwhile, *OsMADS23* in rice plants is reportedly involved in ABA-induced stomata closure [1]. Reports show that ABA accumulation during hypoxic stress is inhibited while the catabolism cascade is increased, and thus, exogenous ABA can increase the tolerance of plants to hypoxic stress [24,25]. Some reports have evaluated that ABA pretreatment of soybean plants enables the plant to withstand flooding due to ABA-induced glycolysis, fermentation, and the tricarboxylic acid cycle [26,27,28].

Furthermore, data from previous reports have predicted that MADS23 can regulate ethylene, which modifies the hypoxic stress response in rice plants. The MADS-box protein SICMB1 has been reported to regulate ethylene biosynthesis in tomato plants [29]. Ethylene is the initial signal for plant adaptation in flooding/hypoxia conditions, which governs ABA, GA, and auxin, all of which affect plant growth and development under hypoxic stress [24,30,31,32]. Moreover, ethylene plays a role in bud elongation, aerenchyma formation, and the development of adventitious roots under flooding/hypoxic conditions [33,34,35]. Mechanistically, the MADS protein is supposed to induce ethylene, inhibit the expression of NCEDs, reduce ABA, and enhance GA, leading to rapid shoot elongation during submergence [36]. Meanwhile, ethylene and its precursor, 1-aminocyclopropane carboxylic acid (ACC), rapidly induce *OsABA8ox1* expression, promoting ABA degradation under flooding, whereas 1-methylcyclopropene (1-MCP) inhibits this effect [37]. Submergence enhances ABA degradation in a Sub1A-independent manner, and exogenous ABA reduces *Sub1* gene expression [38]. This suggests that ABA reduction is crucial for Sub1 accumulation during submergence and highlights the need to explore the mechanisms through which ethylene regulates ABA biosynthesis and signaling under hypoxic stress. Furthermore, adventitious roots are important for coping with hypoxic stress: Adventitious roots can form and grow under water and absorb oxygen more than regular roots. Meanwhile, it has been reported that silencing the MADS-box protein in tomatoes promoted the positive regulation of adventitious roots [39]. Aerenchyma formation is a critical adaptive feature under hypoxic conditions that facilitates oxygen diffusion from shoots to roots. It has been reported that in MADS-box gene transgenic rice plants, the aerenchyma in leaves differentiated earlier than in the wild-type plants. The aerenchyma cavities were also larger in transgenic rice plants than in wild-type plants. These results show that the MADS protein enhances parenchyma development and increases the cavity size [40]. It is assumed that MADS-box transcription factor 23 enhances hypoxia or submergence stress tolerance in plants by regulating anaerobic metabolism, hormone pathways, and morphological adaptations. This integrated response allows plants to survive in low-oxygen environments.

Over the past two decades, researchers have employed various strategies to enhance seedling growth and develop seeds that thrive under flooding and low oxygen conditions. Despite these efforts, progress has been slow due to a shortage of tolerant plant varieties and a limited understanding of the genetic factors involved. Thus, identifying the QTLs or genes associated with seed germination and seedling growth under low oxygen conditions can greatly aid in developing cultivars resistant to low oxygen and flooding. The doubled haploid (DH) method offers significant advantages in plant breeding by enabling the rapid development of completely homozygous lines in a single generation. This technique not only shortens the breeding cycle but also enhances the precision of genetic analysis and quantitative trait loci (QTL) mapping. In rice, DH populations such as the Cheongcheong Nagdong Double Haploid (CNDH) lines provide an ideal resource for studying complex traits, including tolerance to abiotic stresses like hypoxia. The uniform genetic background of DH lines allows for accurate phenotypic evaluation and facilitates the identification of stable QTLs and candidate genes associated with stress tolerance. Therefore, this study aimed to identify rice lines within the CNDH population resistant or susceptible to hypoxic conditions. This study also sought to pinpoint the specific QTLs and genes linked to hypoxia tolerance. This research will contribute to breeding rice varieties that can better withstand environments with low oxygen and flooding.

## 2. Results

### 2.1. Evaluation of Hypoxia-Resistant and Susceptible CNDH Lines

**Figure 1:**
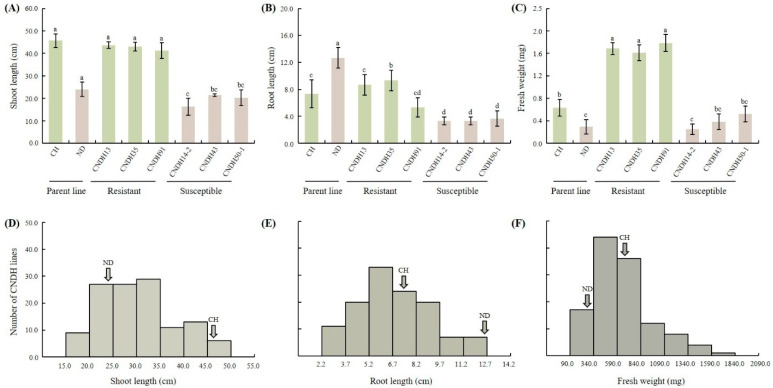
Assessment of plant growth parameters and frequency distribution of shoot length, root length, and fresh weight in the CNDH population. (**A**–**C**) Evaluation of shoot length, root length, and fresh weight. (**D**–**F**) Frequency distribution analysis. Data are shown as the mean ± standard deviation. Different letters above the bars represent a significant difference (*p* < 0.05) as evaluated by Duncan’s multiple range test. CH: Cheongcheong; ND: Nagdong.

**Figure 2:**
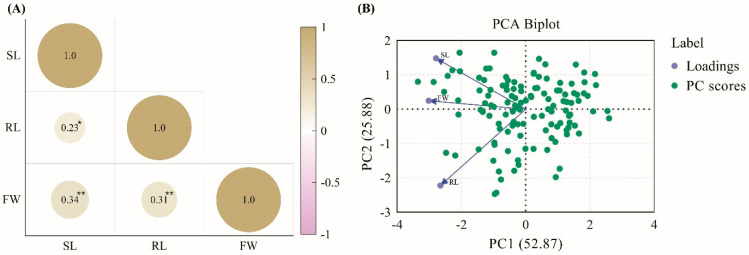
Statistical analysis of shoot length, root length, and fresh weight in the CNDH population. (**A**) Correlation analysis among the investigated traits. (**B**) Principal component analysis (PCA) biplot regarding the three examined traits. ** indicates that the correlation is significant at the 0.01 level, while * correlation is significant at the 0.05 level. SL: shoot length; RL: root length; FW: fresh weight.

This study assessed the responses of 120 CNDH lines and their parent lines to hypoxic conditions. The plants were continuously submerged for two weeks to simulate hypoxic stress. After treatment, the plants were evaluated by measuring shoot length, root length, and fresh weight to assess their resistance or susceptibility to hypoxic conditions. The results indicated that the Cheongcheong parent line exhibited significant resilience under hypoxic conditions, maintaining higher shoot length and fresh weight than the Nagdong parent line, which showed susceptibility with significantly reduced shoot length and fresh weight (Figure 1A,C). Within the CNDH population, lines CNDH13, CNDH35, and CNDH91 demonstrated enhanced shoot length and fresh weight under hypoxic conditions, indicating strong resistance similar to the Cheongcheong parent line, whereas CNDH lines CNDH14-2, CNDH43, and CNDH50-1 exhibited markedly reduced shoot length and fresh weight, paralleling the susceptibility seen in the Nagdong parent line (Figure 1A,C). Interestingly, hypoxic conditions did not significantly impact the root length across resistant and susceptible lines (Figure 1B). The phenotypes based on hypoxia conditions among the CNDH population are listed in Figure S1. The frequency distribution of shoot length, root length, and fresh weight in the CNDH population was assessed using histograms (Figure 1D–F). Normality was further evaluated using the D’Agostino–Pearson and Kolmogorov–Smirnov tests. The results indicated that shoot length and root length followed a normal distribution, whereas fresh weight deviated from normality (Table S1). These observations suggest that they are quantitative in nature and likely regulated by multiple genes. Based on the correlation analysis, all traits exhibited a positive correlation, with no negative correlation observed. In particular, fresh weight demonstrated a highly significant positive correlation with shoot length and root length (Figure 2A). Principal component analysis (PCA) revealed that the first two PCs accounted for 77% of the variance in the dataset. With an eigenvalue of 1.59, PC1 was the primary contributor, representing 52.87% of the total variation and impacting most observed traits. Additionally, PC2 had an eigenvalue of 0.78 and represented 25.88% of the total variability (Figure 2B). Further, the parent lines and selected CNDH lines were categorized into resistant and susceptible groups based on overall plant health under hypoxia. The Cheongcheong and CNDH lines CNDH13, CNDH35, and CNDH91 maintained normal growth and displayed minimal adverse effects from hypoxic stress. In contrast, the Nagdong and CNDH lines CNDH14-2, CNDH43, and CNDH50-1 were significantly affected, with some plants exhibiting severe damage or death (Figure S1). This phenotypic categorization facilitated subsequent gene expression analysis related to hypoxia tolerance, identifying Cheongcheong, Nagdong and CNDH lines CNDH13, CNDH35, CNDH91, CNDH14-2, CNDH43, and CNDH50-1 as promising candidates for further genetic studies and potential breeding programs aimed at enhancing hypoxia tolerance in rice.

### 2.2. Genetic Mapping of Hypoxia-Responsive QTLs

**Figure 3:**
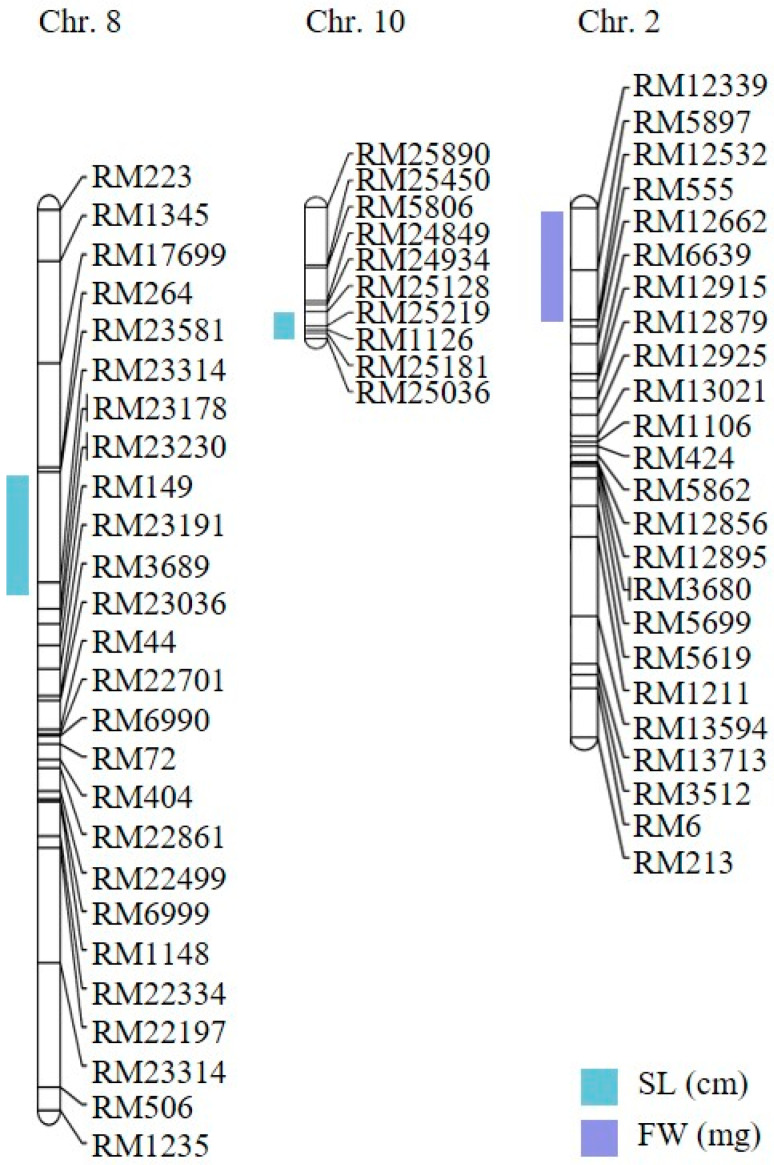
QTL mapping associated with hypoxia in the CNDH population. The QTLs were detected in RM264–RM23314, RM25128–RM25036, and RM12339–RM12532, on chromosomes 8, 10, and 2, respectively. SL: shoot length; FW: fresh weight.

**Figure 4:**
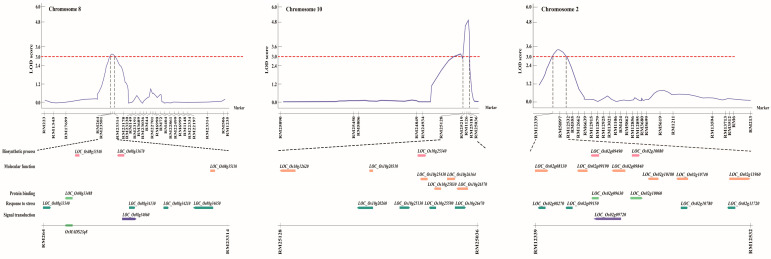
QTL analysis and physical mapping of hypoxia-related genes. Hypoxia-related genes corresponding to biosynthetic process, molecular function, protein binding, response to stress, and signal transduction were identified in RM264–RM23314, RM25128–RM25036, and RM12339–RM12532 on chromosomes 8, 10, and 2, respectively. Among them, *OsMADS23q8* was screened.

To investigate the genetic basis of hypoxia tolerance, we performed a comprehensive QTL analysis using shoot length, root length, and fresh weight as phenotypic traits. In two independent trials, these traits were measured in parent lines and across all CNDH lines. Based on our phenotypic data, we identified three QTLs with a LOD score exceeding 3.0. These QTLs were distributed across three chromosomes: one QTL was detected on chromosome 2, one on chromosome 8, and one on chromosome 10 (Figure 3). Specifically, two QTLs were associated with shoot length (*qSL-8* and *qSL-10*), and one with fresh weight (*qFW-2*). The lack of overlapping QTLs among the identified regions suggests that these traits are not influenced by multiple genomic regions but rather by specific loci. We focused on QTLs with a LOD score higher than 3 to detect candidate genes highly associated with hypoxic stress. Consequently, we selected three QTLs that showed a higher LOD score: *qSL-8* and *qSL-10*, associated with shoot length, and *qFW-2*, related to fresh weight. These QTLs were detected on chromosomes 8, 10, and 2, respectively (Figure 4 and Table 1). The *qSL-8* QTL exhibited a LOD score of 3.02 and was located between markers RM264 and RM23314. The *qSL-10* QTL exhibited a LOD score of 5.03 and was located between markers RM25128 and RM25036. The *qFW-2* QTL exhibited a LOD score of 3.60 and was located between markers RM12339 and RM12532. The phenotypic variation proportion attributable to *qSL-8*, *qSL-10*, and *qFW-2* was 34%, 28%, and 28%, respectively, derived from the alleles of Cheongcheong and Nagdong (Figure 4 and Table 1). These findings provide valuable insights into the genetic loci associated with hypoxia tolerance, potentially guiding future research and breeding programs to improve this trait.

### 2.3. Selection and Relative Expression of Candidate Genes Regulated Against Hypoxia

**Figure 5:**
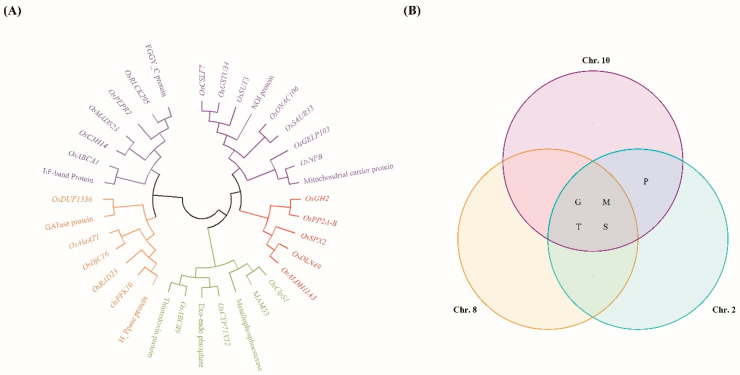
Clustering of candidate genes related to hypoxia. (**A**) A phylogenetic tree was constructed using the genetic distances of each candidate gene, which were divided into five groups and further into subgroups. (**B**) Venn diagram of the candidate genes related to hypoxia. G: gene expression and regulation; M: metabolism and enzymatic activity; P: protein processing, degradation, and modification; T: transport and signal transduction; S: growth, development, and stress response.

To refine our analysis and identify the most promising candidate genes associated with hypoxia tolerance, we specifically assessed QTLs with a LOD score above 3 since this score is recognized as a significant threshold for gene evaluation, providing a robust basis for reducing potential regions of interest. Based on this criterion, we selected three QTLs: one between markers RM264 and RM23314 on chromosome 8, a second between markers RM25128 and RM25036 on chromosome 10, and a third one between markers RM12339 and RM12532 on chromosome 2. Within these regions, we identified 119 genes between markers RM264 and RM23314, 291 genes between markers RM25128 and RM25036, and 263 genes between markers RM12339 and RM12532. These genes were annotated using the NCBI and RiceXpro databases and selected based on their predicted functional annotations. From this selection, nine genes on *qSL-8*, 11 on *qSL-10*, and 15 on *qFW-2* were chosen (Table S3). Figure 5 illustrates the phylogenetic relationships and functional annotations of the selected genes across the three QTLs using a Venn diagram.

**Figure 6:**
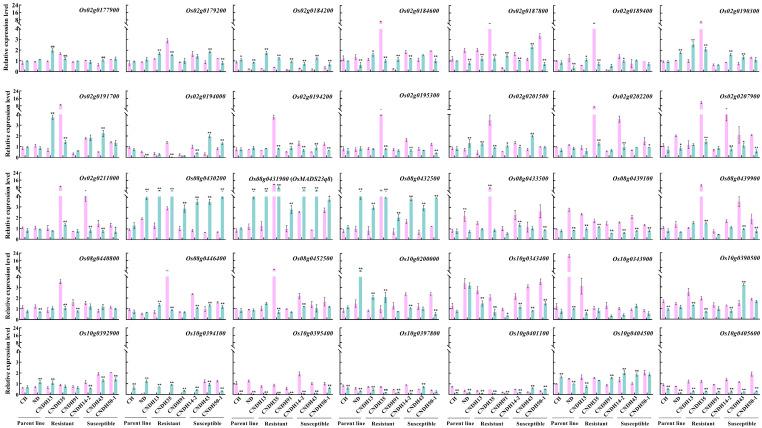
Analysis of the relative expression level of genes related to hypoxia based on QTL analysis. Data are shown as the mean ± standard deviation, and asterisks show a significant difference (* *p* < 0.05, ** *p* < 0.01) following two-way ANOVA and Bonferroni test analysis. Pink bar: control; green bar: hypoxia treatment. CH: Cheongcheong; ND: Nagdong; resistant cell lines: CNDH13, CNDH35, and CNDH91; susceptible cell lines: CNDH14-2, CNDH43, and CNDH50-1.

To investigate the genes most closely associated with hypoxic stress, we determined the relative expression levels of the 35 selected genes (from *qSL-8*, *qSL-10*, and *qFW-2*) by qRT-PCR under hypoxic conditions. This analysis was conducted using parent lines as well as three resistant (CNDH13, CNDH35, and CNDH91) and three susceptible lines (CNDH14-2, CNDH43, and CNDH50-1) (Figure 6). The qRT-PCR results revealed that several genes presented significant changes in expression under hypoxic conditions. Notably, *Os02g0184200*, *Os08g0430200*, *Os08g0431900*, and *Os08g0432500* were significantly upregulated in resistant and susceptible lines under hypoxic stress compared to control plants. Conversely, *Os08g0439100*, *Os10g0343400*, *Os10g0395400*, and *Os10g0405600* were significantly downregulated in resistant and susceptible lines under hypoxic stress compared to control plants. Specifically, the expression of *Os08g0431900* was induced in hypoxic stress compared to control plants in all the lines. The expression pattern and functional annotation of the *Os08g0431900* gene indicate that *Os08g0431900* is highly associated with hypoxic stress. These findings underscore the importance of *Os08g0431900* as a potential target for improving hypoxia tolerance through genetic and breeding strategies. Overall, by focusing on QTLs with high LOD scores and conducting detailed gene expression analyses, we have identified key genetic elements that play crucial roles in the hypoxia response of plants. This approach provides valuable insights for future research and practical applications in enhancing plant hypoxia tolerance.

### 2.4. Phylogenetic, Sequence Homology, and Protein–Protein Interaction Analysis of Selected Genes

The *Os08g0431900* (*OsMADS23q8*) gene was identified due to its transcriptional regulation in rice lines under hypoxic stress. This finding suggests that *Os08g0431900* plays a crucial role in the molecular response to hypoxia. Given the significant roles of *Os08g0431900* in the stress responses, *Os08g0431900* is a promising candidate for further functional characterization and could be a valuable target in breeding programs to improve hypoxia tolerance in rice plants. Hence, we conducted a series of analyses to gain deeper insights into the *Os08g0431900* gene, including phylogenetic studies, sequence homology assessments with other grass species, and protein–protein interaction evaluations using MEGA 11, NCBI, and STRING programs, respectively.

**Figure 7:**
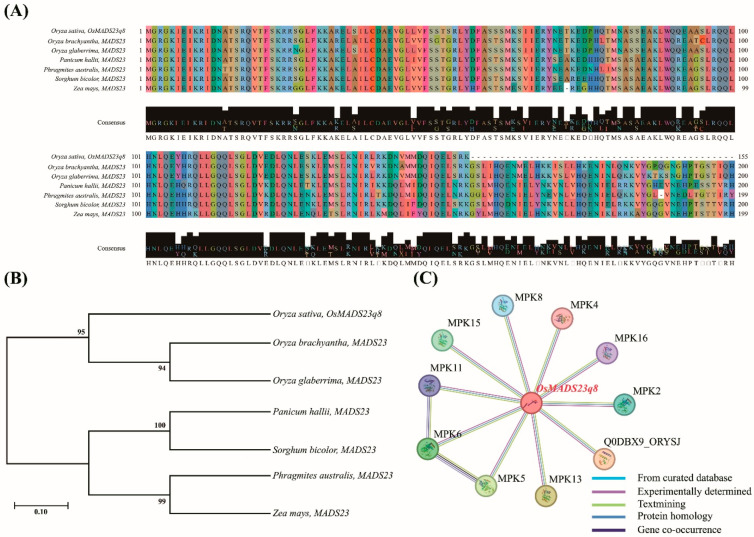
*OsMADS23q8* sequence analysis. (**A**) Multiple sequence alignment of *OsMADS23q8.* A high similarity is illustrated among *Oryza sativa*, *Oryza brachyantha*, *Oryza glaberrima*, *Panicum hallii*, *Phragmites australis*, *Sorghum bicolor*, and *Zea mays*. (**B**) The phylogenetic tree was used to investigate *OsMADS23q8* and the homologous gene. (**C**) *OsMADS23q8* interacts with MPK2, MPK16, MPK4, MPK8, MPK15, MPK11, MPK6, MPK5, MPK13, and Q0DBX9_ORYSJ.

The phylogenetic analysis of *Os08g0431900* revealed a high degree of sequence conservation among several species within the Poaceae family. Notably, *Os08g0431900* shares substantial similarities with homologous genes from *Oryza brachyantha*, *Oryza glaberrima*, *Panicum hallii*, *Phragmites australis*, *Sorghum bicolor*, and *Zea mays* (Figure 7A,B). This conservation suggests that *Os08g0431900* has a functionally important role preserved across these species. Additionally, protein domain analysis showed that *Os08g0431900* exhibits sequence homology with several well-characterized proteins involved in stress responses, such as MPK2, MPK4, MPK5, MPK6, MPK8, MPK11, MPK13, MPK15, MPK16, and Q0DBX9_ORYSJ (Figure 7C).

In summary, the phylogenetic, sequence homology, and protein–protein interaction analyses of *Os08g0431900* underscore its critical roles in the hypoxic stress response. The high degree of sequence conservation and the identified protein interactions support the notion that *Os08g0431900* is functionally significant in stress adaptation. These insights provide a foundation for targeted functional studies to elucidate the precise mechanisms through which *Os08g0431900* contributes to hypoxia tolerance and highlight their potential as targets for genetic improvement efforts to develop rice varieties with enhanced resilience to hypoxic conditions.

## 3. Discussion

This study provides significant insights into the genetic mechanisms underlying hypoxia tolerance in rice, a trait of paramount importance for cultivation in regions prone to flooding. We identified phenotypic markers and genetic loci associated with hypoxia tolerance by evaluating the CNDH rice population and its parent lines under continuous submergence. The observed differential responses, particularly in shoot length and fresh weight, underscore the complexity of hypoxia resistance. Our comprehensive analysis of QTLs pinpointed specific regions on chromosomes 2, 8, and 10, highlighting key genetic determinants. The current study identified 35 hub genes associated with hypoxic stress based on phenotypic evaluations in the QTL analysis. These findings advance our understanding of the genetic basis for hypoxia tolerance and pave the way for targeted breeding strategies to develop resilient rice varieties.

The primary goal of crop breeding technologies is to develop new cultivars better suited to withstand stress conditions. This study evaluated 120 CNDH lines for their response to hypoxic stress induced by deepwater conditions. Based on growth patterns and phenotypic assessments under these conditions, we categorized the CNDH population into resistant and susceptible lines. Among the resistant lines, we identified three highly resistant (CNDH13, CNDH35, and CNDH91) and three highly susceptible lines (CNDH14-2, CNDH43, and CNDH50-1). These selected lines were further evaluated for the expression of specific genes to validate their response to hypoxic stress. Phenotypic assessments revealed that the resistant lines exhibited significantly higher shoot length and fresh weight than the susceptible lines; meanwhile, both the shoot length and fresh weight demonstrated a normal distribution (Figure 1). Similarly, root length was significantly reduced in the susceptible lines compared to the resistant lines, indicating that low oxygen stress negatively affected root development in the susceptible genotypes (Figure 1B). Unlike many other crops, rice plants possess unique adaptive traits that enable them to tolerate submergence. One such trait is the development of aerenchyma. These longitudinally interconnected gas-filled spaces within plant tissues facilitate internal aeration by allowing the transfer of gases between the shoots and roots [41,42,43]. Additionally, rice benefits from leaf gas films, thin layers of air trapped between submerged leaves and surrounding water, which enhance internal aeration during submergence. This adaptation significantly contributes to the ability of the plants to survive and thrive under submerged conditions [44,45,46]. However, many lowland rice cultivars can still succumb to complete submergence, whereby the leaves and stems of these plants only moderately elongate to reach the surface of the water, which often depletes the energy reserves and can lead to death when the flooding becomes too deep and prolonged [47,48]. Some researchers suggest that rice sometimes adopts a strategy of reduced growth to conserve carbohydrates for long-term survival under deepwater conditions [49]. In contrast, our study noted that the resistant CNDH lines (CNDH13, CNDH35, and CNDH91) exhibited increased stem elongation to survive. In contrast, the susceptible lines (CNDH14-2, CNDH43, and CNDH50-1) showed reduced growth and died under prolonged complete submergence. Our results demonstrate that significantly enhanced shoot length during prolonged and complete submergence is a key strategy for hypoxia tolerance in the resistant cell lines, indicating the ability of these plants to survive in deepwater conditions for extended periods. Furthermore, our results showed that the roots of hypoxia-susceptible lines were shorter than those of resistant lines (Figure 1). Several researchers have reported that oxygen supply to the root apex in anaerobic soil during submergence can cause reduced root length [41,42,50,51]. This aligns with our findings, where the reduced root length in susceptible lines under hypoxic conditions highlights the limited ability of these plants to cope with such stress compared to the resistant lines. Overall, our study highlights the importance of shoot elongation as a survival strategy under deepwater submergence and identifies specific CNDH lines that could be valuable for breeding programs to improve submergence tolerance in rice plants.

Additionally, this study identified several QTLs across different chromosomes by focusing primarily on QTLs related to hypoxia tolerance. However, based on a LOD score greater than 3.0, we selected three specific QTLs that are more specifically associated with hypoxic stress: *qSL-8*, *qSL-10*, and *qFW-2*. Furthermore, utilizing predicted functional annotations, we identified 35 genes potentially involved in hypoxic stress responses (Table S3). From the relative expression analysis of these genes, nine showed significant regulation in both resistant and susceptible lines under hypoxic conditions (Figure 6). These genes included *Os02g0184200* (inorganic H+ pyrophosphatase family protein), *Os08g0430200* (Rad23 family protein), *Os08g0432500* (*ClpS* family protein), *Os08g0439100* (Pleckstrin domain-containing protein), *Os10g0343400* (cellulose synthase family protein (*CSLF7*)), *Os10g0395400* (glutathione S-transferase), *Os10g0405600* (phosphofructokinase family protein), and *Os08g0431900* (*MADS23*). Among these, *Os02g0184200*, *Os08g0430200*, *Os08g0432500*, and *Os08g0431900* were significantly enhanced in both the resistant and susceptible lines under hypoxic conditions compared to control plants. The likely reason for the upregulation of these four genes is the presence of hypoxia-responsive elements (HREs) in the promoter regions, which are activated under low oxygen conditions. HREs are specific DNA sequences that bind to transcription factors activated by hypoxia and promote the transcription of genes that help the plant cope with low oxygen levels. Additionally, these genes may play roles in stress response pathways activated under hypoxia, such as anaerobic respiration, which allows plants to produce energy without relying on oxygen. These genes could also be involved in metabolic adjustments that help conserve energy and resources during hypoxic stress, such as switching from aerobic to anaerobic metabolic pathways. Alternatively, the expressions of *Os08g0439100*, *Os10g0343400*, *Os10g0405600*, and *Os10g0395400* were significantly reduced under hypoxic conditions in both the resistant and susceptible lines. Since these genes were downregulated in both cell lines, these data suggest that these genes might not have a direct role in hypoxic stress mitigation. Instead, suppressing these genes could be a strategy to conserve energy for essential survival processes. Hence, downregulating these genes could allow the plant to allocate more resources towards critical functions necessary for surviving low oxygen conditions. While both the resistant and susceptible lines show similar gene expression and suppression trends under hypoxic conditions, the resistant lines likely possess additional mechanisms or more efficient gene regulation processes that confer greater tolerance. However, the exact functions of these genes in response to hypoxia in rice have yet to be fully elucidated and require further molecular, physiological, anatomical, and metabolic assessment. Understanding these mechanisms in greater detail could provide valuable insights into improving hypoxia tolerance in rice and other crops.

Our study identified *Os08g0431900* (MADS23 transcription factor) as a key gene among nine differentially regulated genes in resistant and susceptible CNDH lines under hypoxic stress based on its expression pattern and functional annotation; these data are consistent with previously published studies. *OsMADS23* promotes the accumulation of endogenous ABA and proline by activating the transcription of key biosynthetic genes, including *OsNCED2*, *OsNCED3*, and *OsNCED4* for ABA, and *OsP5CR* for proline [17]. Consistent with previous QTL-meta analyses identifying *Os02g0304900*, *Os01g0568400*, and *Os01g0566500* as regulators of ABA metabolism and signaling during anaerobic germination [52], our study highlights *MADS23* as a candidate gene modulating the ABA pathway, suggesting that ABA-mediated signaling is a conserved mechanism underlying hypoxia tolerance in rice seedlings. *Os08g0431900* was significantly induced under hypoxic stress in both the susceptible and resistant lines (Figure 6). To elucidate the role of *Os08g0431900* during hypoxic stress, it is essential to understand the mechanism through which the MADS family genes mitigate hypoxic stress. The MADS-box gene family comprises transcription factors characterized by a conserved DNA-binding domain known as the MADS-box. These genes are widely distributed across various living organisms and play diverse functional roles. MADS-box genes are classified into two major evolutionary lineages in plants: type I and type II [53,54]. Approximately 100 functionally active MADS-box genes have been identified in Arabidopsis and around 70 in rice [55]. *OsMADS26* has been reported to trigger multiple responses associated with various stress conditions and roles in flower development, vegetative growth, and root development [56]. Interestingly, the cell lines overexpressing *OsMADS26* demonstrated an increased expression of genes involved in ethylene biosynthesis, suggesting a potential role of *OsMADS26* in regulating ethylene-related pathways [56]. It has also been reported that SICMB1 (MADS-box protein) regulates ethylene biosynthesis in tomato plants [29]. Ethylene promotes hypoxia tolerance in Arabidopsis [57] and acts as a primary signal for plant adaptation to flooding and hypoxic conditions, regulating key hormones such as ABA, GA, and auxins, which collectively modulate plant growth and development under these stress conditions [24,30,31,32]. Recently, integrating GWAS and transcriptome data identified five genes such as, LOC_Os01g07420, LOC_Os02g01890, LOC_Os03g45720, LOC_Os04g56920, and LOC_Os11g41680, potentially associated with rice hypoxic germination tolerance, with LOC_Os11g41680 encoding a cytochrome P450, and such genes are known to regulate plant height via brassinosteroid pathways acting synergistically with gibberellins and auxins [58,59]. Hypoxia-tolerant rice has higher levels of IAA, and applying IAA to sensitive varieties improves their survival, showing that IAA helps seedlings grow and cope with low-oxygen stress [60]. Additionally, ethylene is critical in promoting bud elongation, aerenchyma formation, and the development of adventitious roots; these processes are essential for plant survival in response to flooding and hypoxia [33,34,35]. Supporting this, a recent study on brassinosteroids in submerged rice found that among the differentially expressed genes (DEGs), several ethylene synthesis genes, along with GA oxidation and JA synthesis genes, were identified, highlighting the central role of ethylene in regulating coleoptile elongation under hypoxic conditions [61]. This study did not quantify ethylene levels due to resource limitations. However, qRT-PCR analysis indicated that rice plants may induce ethylene production through the upregulation of the *MADS23* gene, suggesting a potential regulatory role of this gene in ethylene biosynthesis under hypoxic conditions. Additionally, ethylene stabilizes the ethylene-responsive factors (ERFs) [62], which are crucial in mitigating hypoxic stress. Consistent with our findings, several studies have reported the involvement of ERFs under hypoxic/low oxygen stress [3,63,64]. It was previously reported that rice varieties cultivated in deep paddies induce the ERF transcription factor, enabling rapid stem elongation in slowly established foods [65]. Recent studies have shown that the ERF transcription factor family is pivotal in regulating gene expression under low oxygen conditions, redundantly activating a range of hypoxia-responsive genes through direct promoter interaction [66,67,68]. For instance, overexpression of the *ERF* family member *RAP2.12* induced the primary hypoxic transcriptional response in Arabidopsis [69]. Under hypoxic conditions, ERF–VII proteins stabilize and initiate the transcription of anaerobic genes by binding to the hypoxia-responsive promoter element (HRPE) in the promoter regions of these genes [68]. The fermentative genes *PDC1* and *ADH* are activated anaerobic genes [64]. The transcriptional activation of these fermentation pathways, along with glycolysis, ensures the production of minimal ATP necessary for essential cellular functions, thereby maintaining cell integrity and viability under hypoxic conditions [70].

Overall, our research highlights critical genetic and phenotypic adaptations that confer hypoxia tolerance in rice plants. Identifying key QTLs and the expression of *Os08g0431900* (MADS23 transcription factor) offer new insights into the mechanisms involved in hypoxia resistance. These findings suggest that MADS23 may be involved in ethylene biosynthesis, which plays a crucial role in plant adaptation to hypoxic stress by regulating key hormones, aerenchyma formation, and root development. Furthermore, the regulation of ethylene-responsive factors underscores the importance of these factors in mitigating hypoxic stress, with ERF transcription factors activating hypoxia-responsive genes to promote anaerobic metabolism and ensure plant survival. This insight expands our understanding of how the MADS-box protein and ethylene signaling contribute to stress tolerance mechanisms in rice, providing a foundation for future studies to enhance crop resilience under suboptimal environmental conditions.

## 4. Materials and Methods

### 4.1. Plant Material and Experimental Design

The CNDH population was developed using a doubled haploid from a cross between Cheongcheong (Indica) and Nagdong (Japonica), originating from the F_1_ hybrid anther culture [71]. To identify hypoxia-resistant and susceptible lines within the CNDH population, we initially screened the entire population under hypoxic conditions. The experiment used 50 trays in a greenhouse maintained under optimal environmental conditions. Before planting, seeds were sterilized using Spotak pesticide (Hankooksamgong, Seoul, South Korea) and soaked for three days at 33 °C in an incubator, following a previous method [72]. After successful germination, the seeds were transferred to the trays, with ten seedlings grown per hole for each line. Two-week-old seedlings were subjected to hypoxic stress through submersion in a 30 cm deepwater tub for two weeks. Hypoxia has previously been reported to regulate the shoot length, root length, and fresh weight of the *Medicago truncatula* plant [73]. Therefore, measuring shoot length, root length, and fresh weight in rice under hypoxic conditions is essential to assess the physiological adaptations performed by each plant, such as shoot elongation for gas exchange and root development for nutrient uptake, both of which are impaired under low oxygen. Moreover, identifying the QTLs associated with these traits can aid in breeding rice varieties that are better adapted to hypoxic stress, ultimately improving survival and yield in flood-prone or waterlogged environments.

### 4.2. Construction of a Genetic Map and Analysis of QTLs Following Hypoxia

A total of 778 simple sequence repeat (SSR) markers were used to generate the genetic map of the CNDH population. A total of 423 SSR markers among the 778 SSR markers observed demonstrated a polymorphism, with 222 screened further using PCR amplification of the co-dominant genes [71,74]. The genetic map for the CNDH population covered a total distance of approximately 2112.7 cM, with an average marker spacing of 10.6 cM [75]. QTL analysis for hypoxia tolerance was conducted using Win QTL Cartographer 2.5 software (North Carolina State University, Raleigh, NC, USA). This software requires input of the genetic distances between markers, marker names, chromosome numbers, genotypic data, and target trait values. Composite Interval Mapping (CIM) was performed across the genome using a logarithm of the odds (LOD) score threshold set at 3.0 to ensure statistical significance in the QTL detection. Identified QTLs were named following the method proposed by McCough [76].

### 4.3. Annotation of Candidate Genes Related to Hypoxia

The identified QTLs were further examined for potential candidate genes using the RiceXpro database (https://ricexpro.dna.affrc.go.jp) and RAP-DB (https://rapdb.dna.affrc.go.jp), accessed in March 2024. Several candidate genes were identified between the two markers. Gene functions were annotated through Gene Ontology (GO) analysis, utilizing the resources provided by the Rice Genome Annotation Project (http://rice.uga.edu/index.shtml), also accessed in March 2024. To analyze homologous sequences, we employed NCBI (https://www.ncbi.nlm.nih.gov) and Jalview 2.11.2.0 (https://www.jalview.org), with access in April 2024. Phylogenetic trees were constructed using MEGA 11 (https://www.megasoftware.net), and protein–protein interactions were assessed using STRING version 11.0 (https://string-db.org).

### 4.4. Relative Gene Expression

Quantitative real-time PCR (qRT-PCR) was performed to assess the expression levels of the selected candidate genes. Based on average shoot length, average root length, average fresh weight (three replicates of each single line), and overall plant health, three hypoxia-resistant rice lines (CNDH13, CNDH35, and CNDH91) and three hypoxia-susceptible rice lines (CNDH14-2, CNDH43, and CNDH50-1) were chosen, along with the parental lines Cheongcheong and Nagdong. These selected lines were grown under the same previously described hypoxic conditions. For RNA extraction, three leaves from three plants of each line were collected after two weeks of hypoxia treatment. RNA was isolated using the RNeasy Plant Mini kit (Qiagen, Hilden, Germany) following the manufacturer’s instructions. Complementary DNA (cDNA) was synthesized using the UltraScript 2.0 cDNA Synthesis kit (PCRBIOSYSTEM, Wayne, PA, USA) per the provided guidelines. qRT-PCR was performed using the StepOnePlus Real-Time PCR System (Fisher Scientific, Hampton, NH, USA) with 2X Real-time PCR Master Mix (including SYBR^®^ Green I) (BIOFACT, Daejeon, Korea). The amplification conditions were as follows: polymerase activation at 95 °C for 10 min, denaturation and annealing at 95 °C for 15 s, and extension at 60 °C for 1 min. *OsActin* was used as the reference (housekeeping) gene, and relative expression levels were calculated using the 2^−∆∆CT^ method [77]. The qRT-PCR assays were conducted in triplicate to determine the average and standard deviation. The primers used for the selected genes are listed in Table S2.

### 4.5. Statistical Analysis

To assess the significance of the differences between the means of the replicates, Duncan’s multiple range test (DMRT) using SPSS (IBM SPSS Statistics, version 25, Redmond, WA, USA) and two-way ANOVA were conducted using GraphPad Prism software (version 8.0.2, Dotmatics, San Diego, CA, USA). Statistical significance is as follows: * indicates a *p* < 0.05, and ** represents a *p* < 0.01, reflecting the significance of differences among the three biological replicates.

## 5. Conclusions

Our study elucidates the genetic mechanisms underpinning hypoxia tolerance in rice plants, which is vital for cultivation in flood-prone areas. By assessing the CNDH rice population and its parent lines under continuous submergence, we identified phenotypic markers and genetic loci associated with hypoxia tolerance, specifically on chromosomes 2, 8, and 10. The differential expression of candidate genes, particularly *Os08g0431900*, and their conservation across related species underscore their potential roles in the hypoxia response. Our findings suggest enhanced shoot elongation is a critical strategy for hypoxia resistance, with resistant lines displaying significantly greater shoot length and fresh weight than susceptible lines. Expression analysis revealed nine genes significantly regulated under hypoxic conditions. Among these, *Os08g0431900* was significantly expressed under hypoxic stress, indicating its involvement in hypoxic stress mitigation. This study advances our understanding of hypoxia tolerance in rice by identifying key genetic determinants and adaptive traits. These insights can provide a valuable framework for targeted breeding strategies to develop rice varieties resilient to flooding and hypoxic stress, ensuring sustainable rice production in vulnerable regions.

## Figures and Tables

**Table 1:** QTLs associated with hypoxia in the CNDH population.

Traits	QTLs	Chr	Interval Markers ^z^	LOD	Additive Effect ^y^	R^2 x^	Increasing Effects ^w^
Shoot length (cm)	*qSL-8*	8	RM264–RM23314	3.02	4.24	0.34	Cheong Cheong
	*qSL-10*	10	RM25128–RM25036	5.03	6.39	0.28	Cheong cheong
Fresh weight (mg)	*qFW-2*	2	RM12339–RM12532	3.60	−0.12	0.28	Nagdong

SL: shoot length; FW: fresh weight. ^z^ The markers in the significance threshold. ^y^ The positive values indicate the contribution from the mother plant. ^x^ The phenotypic variation. ^w^ The source of the allele generating an increase in assessed traits.

## Data Availability

The original contributions presented in this study are included in the article/Supplementary Materials. Further inquiries can be directed to the corresponding author.

## Supplementary Materials

The following supporting information can be downloaded at: https://www.mdpi.com/article/10.3390/ijms262110420/s1.
