# Supplementary material for: Identification of Quantitative Trait Loci and Candidate Genes Involved in Rice Seedling Growth Under Hypoxic Stress
Source: Int J Mol Sci. 2025 Oct 27;26(21):10420. doi: 10.3390/ijms262110420 (PMC12610234; doi:10.3390/ijms262110420)
Supplement: Supplementary file 1 [file ijms-26-10420-s001.zip › ijms-3942154-supplementary.pdf]

Supplementary Materials

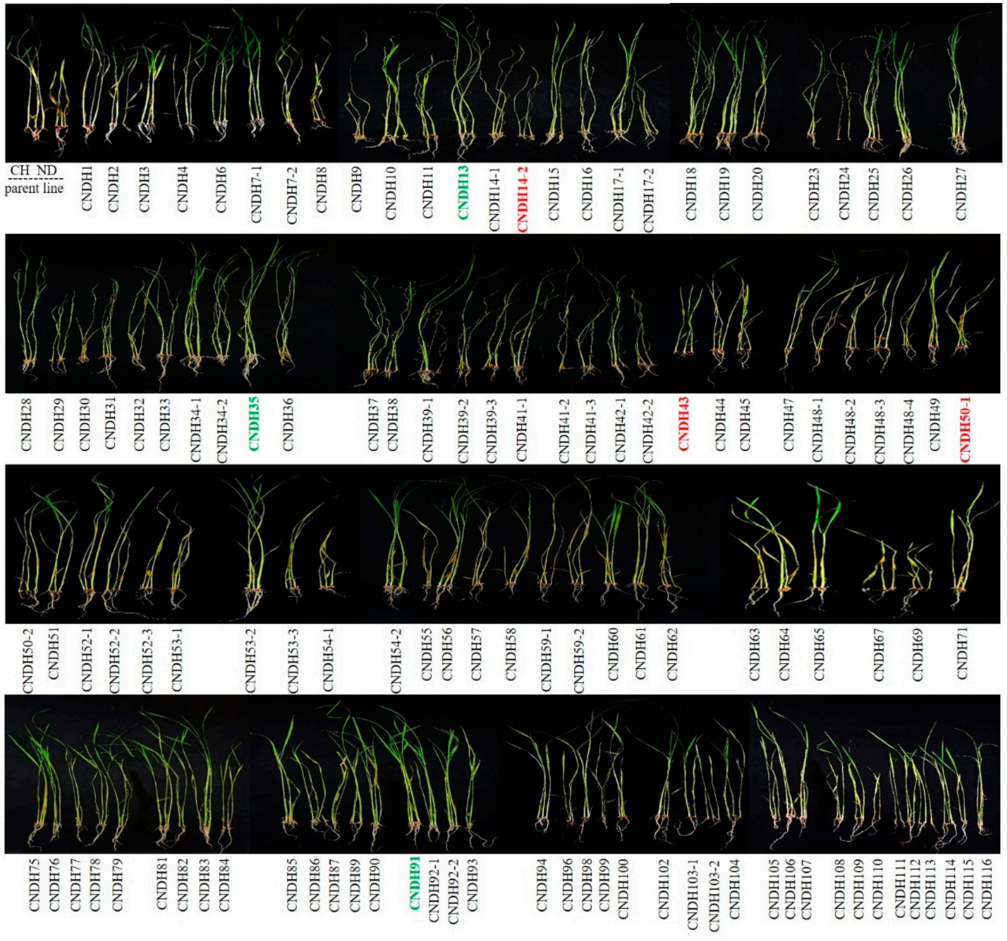

Figure S1. Phenotypes based on hypoxia conditions among the CNDH population. CH: Cheongcheong. ND: Nagdong. Red color: Susceptible lines. Green color: Resistant lines

Table S1 Normality tests (D'Agostino–Pearson and Kolmogorov–Smirnov).

Traits	Kolmogorov-Smirnov (KS)	p-value	D'Agostino–Pearson (K2)	p-value	Distribution	Mean	Median	Mode
SL	0.0597	<.100	4.62	0.099	Normal	30.6	30	29.3
RL	0.0741	0.097	4.05	0.132	Normal	6.8	6.67	7.3
FW	0.0986	0.005	18.5	<.001	Non-normal	0.59	0.54	0.35

Table S2 Primer set for qRT-PCR analysis.

Primer Name	Forward<br>Reverse	Sequence (5' to 3')
OsActin	F	TGAATCTGGTCCAGGCATCG
	R	TGGGACGCATGCAAACAATC

Os02g0177900	F	GGCTTGGGAGACCTTGTTGA
	R	CAGCAGTAGCTGTCAACCCA
Os02g0179200	F	GTCGAGCATTGGGTGGTAGA
	R	GACCCCTCAAAATCACGCAC
Os02g0184200	F	CTTGGTCCCAAAGGTTCCGA
	R	GGCCTTTGTTACACCCAACG
Os02g0184600	F	AGGACATCTTGGACGTGCTG
	R	CGCGTTGATGAGCACGTTAG
Os02g0187800	F	TCATCTCGCCCATGGTGATG
	R	GCCTGGTTGACGTAGTCCAT
Os02g0189400	F	ACCCTGTTGAGTTTTGCTTGC
	R	AATGATGGCAGGACAGGACA
Os02g0190300	F	CGTACCACGTTGACCTTGGA

	R	TGTCACACTTGGAGACAGCA
Os02g0191700	F	CTTCCTCATCCGCCAAATGC
	R	CCCAAACATCTCCACTTGCG
Os02g0194000	F	AGTCCTCTTTGCCGATTGGG
	R	AAGACCTGGCCGGTAATTGG
Os02g0194200	F	TCAAACCGGTGTAGGAAGCA
	R	AAGTGGCAACCCTCACCAAA
Os02g0195300	F	CATGGTGGCGATGGCG
	R	ATAGTCACCCTCCGCTTCCT
Os02g0201500	F	TCACCTTCCTTGAAGCTGCC
	R	CAGTGGGTCACCAGAAGACG
Os02g0202200	F	AAGCTCGTGAAGGAATGCGA
	R	TGGGCTTTGAACCCTTCTCC

Os02g0207900	F	GGTACATGTCGTCGGGGAAG
	R	AATGAGCTCACTGCTCACCC
Os02g0211000	F	CTCCGTGGCCTGATCAGTAA
	R	CCCCTGCTCATAGCTCATCC
Os08g0430200	F	CACCTGTTAGCGAGCCTGT
	R	CAGGAACCCCGGTGTACAAA
Os08g0431900	F	TGCTTGACACTGCACAAACT
	R	TCTTGATGGACAAGGCTGACT
Os08g0432500	F	GAGGCTCATGTAAACGGGCT
	R	GCAGAGCCTCTTCTTCAGCA
Os08g0433500	F	TCGGTGACTGTAATGGTAGTGA
	R	CGCTCTGAGCTCCTCTGATG
Os08g0439100	F	ATCCGTTGCACGGAGCATTA

	R	TCGCACGATTTAGGCGAACT
Os08g0439900	F	AGGATGCACATCAGCGTCAA
	R	CAACTCACTGAAATGGCGGC
Os08g0440800	F	CAAGACCCCGATTGCAGAGT
	R	GGCCAATCTTGGAGACTGCT
Os08g0446400	F	CAGACAAAGGAGCATAATGGGA
	R	GAGCTGGGCAGTATCAGAGTC
Os08g0452500	F	CGAAGTGAAGAGTACGAAGCG
	R	ATTCCCCAATTAGCACCGACG
Os10g0200000	F	TGCAAGCACAACAAGGGGTA
	R	ACTGGCCATCGAAACACAGA
Os10g0343400	F	CGACTCGCATCTCGACAACT
	R	GCAGCGTAGGTGATTCTCCA

Os10g0343900	F	GCTGGGGTTACATCATGGCT
	R	ATTGCCGGCAGTCTGGAAAA
Os10g0390500	F	CATCGCGTTTTTGCGGTTTT
	R	CTCTCCACGCACTGCATACT
Os10g0392900	F	TTACACGGAAGCGGTGAACA
	R	TGGGTTTGGGCATCGGTATT
Os10g0394100	F	TGGCCCGTTAGCTTGTGAAT
	R	CGCTCGATACCATATTCGACTG
Os10g0395400	F	CGAGGTTCCTCCAAACCAAGT
	R	GAGCGATCGAACTTTGGGAC
Os10g0397800	F	AGGCATCGTAGCTGTAACCA
	R	CAATGTGAAACGCAGATGCT
Os10g0401100	F	ATATGCTTCCAGGTGCCAGG

	R	CTGCTGCTTGAAGTTGGCTG
Os10g0404500	F	TGACAGTGACAGAGCTTGATGA
	R	GAGAAGTCGAGCAGCCAGAA
Os10g0405600	F	GCATTGACCCTGATTGCAGC
	R	TTGAGACCAGGGCAAAGTCC

Table S3 Hypoxia related genes on chromosome 8, 10, and 2.

Chromosome	Marker interval	Locus	Description
8	RM264-RM23314	*Os08g0430200*	UV excision repair protein Rad23 family protein
		Os08g0431900	Transcription factor MADS23

		Os08g0432500	ATP-dependent Clp protease adaptor protein ClpS family protein
		Os08g0433500	No apical meristem (NAM) protein domain containing protein
		Os08g0439100	Pleckstrin homology-type domain containing protein
		Os08g0439900	Mitochondrial glycoprotein family protein
		Os08g0440800	Glyceraldehyde-3-phosphate dehydrogenase
		Os08g0446400	Leucine rich repeat, N-terminal domain containing protein
		Os08g0452500	Auxin responsive SAUR protein family protein
10	RM25128-RM25036	*Os10g0200000*	Protein kinase-like domain containing protein
		Os10g0343400	Cellulose synthase family protein
		Os10g0343900	Thioredoxin fold domain containing protein

		Os10g0390500	Alanine aminotransferase
		Os10g0392900	Lipolytic enzyme, G-D-S-L family protein
		Os10g0394100	Metallophosphoesterase domain containing protein
		Os10g0395400	Glutathione S-transferase, N-terminal domain containing protein
		Os10g0397800	Mitochondrial carrier protein family protein
		Os10g0401100	Zinc finger, RanBP2-type domain containing protein
		Os10g0404500	Sucrose transporter
		Os10g0405600	Phosphofructokinase family protein
2	RM12339-RM12532	*Os02g0177900*	Carbohydrate kinase, FGGY family protein
		Os02g0179200	Glutamine amidotransferase class-I domain containing protein

Os02g0184200	Inorganic H+ pyrophosphatase family protein.
Os02g0184600	Cytochrome P450 family protein
Os02g0187800	Cinnamyl alcohol dehydrogenase (EC 1.1.1.195)
Os02g0189400	Nitrate-induced NOI family protein
Os02g0190300	ABC transporter related domain containing protein
Os02g0191700	Calcium-binding EF-hand domain containing protein
Os02g0194000	Myb, DNA-binding domain containing protein
Os02g0194200	Zinc finger, CCCH-type domain containing protein
Os02g0201500	EF-Hand type domain containing protein
Os02g0202200	SPX, N-terminal domain containing protein

Os02g0207900	Alpha/beta hydrolase family protein
Os02g0211000	ABC transporter related domain containing protein
